# Increased hyperpolarized [1‐^13^C] lactate production in a model of joint inflammation is not accompanied by tissue acidosis as assessed using hyperpolarized ^13^C‐labelled bicarbonate

**DOI:** 10.1002/nbm.3892

**Published:** 2018-01-30

**Authors:** Alan J. Wright, Zoé M.A. Husson, De‐En Hu, Gerard Callejo, Kevin M. Brindle, Ewan St. John Smith

**Affiliations:** ^1^ Cancer Research UK Cambridge Institute University of Cambridge, Li Ka Shing Centre, Robinson Way Cambridge UK; ^2^ Department of Pharmacology University of Cambridge, Tennis Court Road Cambridge UK; ^3^ Department of Biochemistry University of Cambridge, Tennis Court Road Cambridge UK

**Keywords:** acidosis, arthritis, inflammation, lactate, MRI, pain

## Abstract

Arthritic conditions are a major source of chronic pain. Furthering our understanding of disease mechanisms creates the opportunity to develop more targeted therapeutics. In rheumatoid arthritis (RA), measurements of pH in human synovial fluid suggest that acidosis occurs, but that this is highly variable between individuals. Here we sought to determine if tissue acidosis occurs in a widely used rodent arthritis model: complete Freund's adjuvant (CFA)‐induced inflammation. CFA robustly evoked paw and ankle swelling, concomitant with worsening clinical scores over time. We used magnetic resonance spectroscopic imaging of hyperpolarized [1‐^13^C]pyruvate metabolism to demonstrate that CFA induces an increase in the lactate‐to‐pyruvate ratio. This increase is indicative of enhanced glycolysis and an increased lactate concentration, as has been observed in the synovial fluid from RA patients, and which was correlated with acidosis. We also measured the ^13^CO_2_/H^13^CO_3_^−^ ratio, in animals injected with hyperpolarized H^13^CO_3_^−^, to estimate extracellular tissue pH and showed that despite the apparent increase in glycolytic activity in CFA‐induced inflammation there was no accompanying decrease in extracellular pH. The pH was 7.23 ± 0.06 in control paws and 7.32 ± 0.09 in inflamed paws. These results could explain why mice lacking acid‐sensing ion channel subunits 1, 2 and 3 do not display any changes in mechanical or thermal hyperalgesia in CFA‐induced inflammation.

## 1 INTRODUCTION

Arthritis underlies about 40% of cases of chronic pain,^1^ and understanding the cause of this pain is essential in developing new analgesics. During inflammation, sensory neurones innervating joints become sensitized,^2^, ^3^, ^4^, ^5^ which contributes to the tenderness of arthritic joints.^6^, ^7^ The inflammatory milieu contains many mediators, including protons, and early measurements of synovial fluid pH from rheumatoid arthritis (RA) patients found a pH of 7.21, compared with 7.43 in healthy subjects.^8^ Similar acidosis was observed by others,^9^, ^10^ with a fourth study showing that RA synovial fluid can become very acidic (pH 6.61 versus pH 7.30).^11^ However, a more recent study found that, although RA synovial fluid was more acidic than that of synovial fluid from osteoarthritis (OA) patients, neither was acidic (RA pH ~ 7.5 and OA pH ~ 7.65).^12^ Moreover, the reported pH range shows clear individual variation: pH ~ 6.84‐7.43,^9^ and pH ~ 6.95‐7.75,^12^ suggesting that acidosis is not necessarily a common feature of arthritis. Several studies have found a correlation between synovial lactate concentration and acidosis,^9^, ^12^, ^13^ and it has been suggested that acidosis results from inflammation, where glucose is metabolized to pyruvate and then to lactic acid under the relatively anaerobic conditions of arthritic synovial fluid, where low pH and lactic acid concentrations correlate with low O_2_ concentration.^9^, ^10^, ^14^

Acid evokes pain in humans,^15^, ^16^, ^17^ and thus inhibiting acid‐induced activation of sensory neurones is an appealing strategy for treating arthritic pain. Sensory neurones express several acid sensors: acid‐sensing ion channels (ASICs), transient receptor potential vanilloid 1 (TRPV1), proton‐sensing GPCRs and certain background K^+^ channels.^18^ Furthermore, response to low pH has been shown to be sensitized by inflammatory mediators.^19^, ^20^, ^21^, ^22^, ^23^, ^24^

We and others have demonstrated expression and function of TRPV1 and ASICs within articular sensory neurones,^25^, ^26^, ^27^ and both TRPV1 and ASIC3 are upregulated in different inflammatory models, suggesting their involvement in inflammatory pain.^26^, ^28^, ^29^ A common model for inflammatory arthritis involves intraplantar/intraarticular injection of complete Freund's adjuvant (CFA),^30^ which although not replicating autoimmunity does provide a robust model of RA‐like arthritis: T cell‐mediated pathogenesis, leukocyte invasion, synoviocyte hyperplasia, pannus formation and disrupted gait.^30^, ^31^, ^32^ However, it is unknown if tissue acidosis occurs in this commonly used model. In CFA studies investigating animal pain behaviour, TRPV1 knockout mice show diminished thermal hyperalgesia, but no change in mechanical hyperalgesia,^33^ and animals lacking either ASIC1, ASIC2 or ASIC3 show no alleviation of thermal/mechanical hyperalgesia.^34^

Hyperpolarization of ^13^C nuclei can increase their sensitivity to detection in a magnetic resonance experiment by more than 10 000‐fold.^35^ This enormous increase in sensitivity has enabled real‐time imaging of tissue metabolism *in vivo* following intravenous injection of hyperpolarized ^13^C‐labelled substrates,^36^ including in humans.^37^, ^38^ Previous magnetic resonance spectroscopic imaging (MRSI) studies in rats injected with hyperpolarized [1‐^13^C]pyruvate showed raised lactate‐to‐pyruvate ratios in CFA‐induced inflammation,^39^ which, considering the relationship observed between lactic acid and acidosis,^9^, ^12^, ^13^ suggested that acidosis must occur in this widely used model. The aim of this study was to determine if tissue acidosis actually does occur in regions where there was increased lactate labelling by using hyperpolarized [^13^C]bicarbonate to measure tissue extracellular pH.^40^, ^41^

## 2 MATERIALS AND METHODS

### 2.1 Animals

All experiments were conducted in accordance with the UK Animal (Scientific Procedures) Act 1986 Amendment Regulations 2012 under a Project License (70/7705) granted to E. St. J. S. by the Home Office; the University of Cambridge and Cancer Research UK Cambridge Institute animal welfare ethical review bodies also approved procedures. Female C57BL/6 mice (aged 10‐12 weeks and weighing 18‐20 g, Envigo, Huntingdon, Cambridgeshire, UK) were housed in groups of up to four mice per cage with nesting material and a cardboard tube; the holding room was temperature controlled (21°C) and mice were on a standard 12 h light/dark cycle with food and water available *ad libitum*.

All chemicals were purchased from Sigma‐Aldrich (Gillingham, Dorset, UK), unless stated otherwise.

### 2.2 CFA‐induced inflammation

Mice were anaesthetized using isofluorane (2%), and two 15 μl injections of 10 mg/ml CFA (Chondrex, Redmond, WA, USA) were made using a Hamilton syringe and 27 G needle, to give a total dose of 300 μg per paw. Control injections with phosphate‐buffered saline (PBS) were made in the contralateral hind paw.

### 2.3 Assessment of paw swelling and clinical scores

Mice were weighed and calliper measurements of ankle and foot pad diameters were performed daily. Clinical scores were also made daily according to Reference 42, with a score of 0 for a normal paw, 1 for a slight swelling and/or erythema, 2 for a pronounced swelling and 3 for ankylosis of the paw and ankle. A two‐way ANOVA was used to compare changes in calliper measurements and clinical scores between CFA‐ and PBS‐injected paws over time (*n* = 5 mice from days 1 to 5). Sidak's multiple comparison test was used to compare each time point.

### 2.4 MRI

Images and spectra were acquired using a 7 T MR instrument (Agilent, Palo Alto, CA, USA). Proton images were acquired axially through downwards pointing feet using a volume coil. Images were acquired using a fast spin echo (FSE) sequence (40 × 40 mm^2^ slices, 2 and 6‐10 mm thick, covering the entire foot, 128 × 128 data points, eight echoes, effective echo time (*T*_E_) of 48 ms, 2 s repetition time (*T*_R_)) or using a spoiled gradient echo (GE) sequence (40 × 40 mm^2^, 2 mm thick slices; 128 × 128 data points; *T*_R_, 400 ms; *T*_E_ 2.85 ms). Proton imaging was used to confirm positioning and to quantify foot volumes.

### 2.5 Magnetic resonance spectroscopic imaging of hyperpolarized [1‐^13^C]pyruvate metabolism

^13^C images were acquired from both non‐inflamed hind paws of two mice following injection of hyperpolarized [1‐^13^C]pyruvate 1 day prior to injection with CFA and PBS. CFA and PBS were then injected, as described above, and inflammation allowed to develop for five to seven days before the ^13^C imaging was repeated. Each mouse was placed inside a ^1^H/^13^C volume coil (bird cage, internal diameter 42 mm) with both feet arranged vertically inside a 20 mm circular ^13^C receive coil (RAPID Biomedical, Rimpar, Germany). There was no significant difference in body weight between mice with CFA‐inflamed and non‐inflamed paws at the time of imaging (mean 18 g). A reference FSE proton image was acquired as a single axial slice (6‐10 mm thick) that encompassed both feet including the heel, up to where the top of the talus meets the leg. [1‐^13^C]pyruvate (99% ^13^C labelled, 44 mg) in a solution containing 15 mmol/l of trityl radical, tris(8‐carboxy‐2,2,6,6‐tetra(hydroxyethyl)‐benzo‐(1‐5)‐bis‐(1,3)‐dithiole‐4‐yl)‐methyl sodium salt (OX063; GE Healthcare, Amersham, UK) and 1.5 mmol/l gadolinium chelate (Dotarem, Guerbet, Paris, France) was polarized as described previously^43^ using a Hypersense dynamic nuclear polarization (DNP) system (Oxford BioTools, Abingdon, UK). The sample was dissolved in superheated (180°C, ~10 bar) buffer (40 mM 4‐(2‐hydroxyethyl)‐1‐piperazine‐ethanesulfonic acid (HEPES), 94 mM NaOH, 30 mM NaCl and 50 mg/l ethylenediaminetetraacetic acid (EDTA)) to give a final pyruvate concentration of 0.82 M, 200 μl of which was injected via a tail vein. Magnetic resonance spectroscopic images (32 × 32 voxels, 40 × 40 mm^2^ field of view, 6‐10 mm thick slice) were acquired from the same spatial location as the reference proton image. The sequence was started 20 s after pyruvate injection and had a nominal flip angle of 5°, an echo time of 0.85 ms and a repetition time of 30 ms to give a total acquisition time of 31 s. MRSI data were processed in MATLAB (MathWorks, Natick, MA, USA) using custom‐written scripts. The data were Fourier transformed and individual voxel free induction decays were modelled^44^ with four resonances using a least squares fitting routine. The amplitudes of the resonances at the chemical shifts of [1‐^13^C]pyruvate (173 ppm) and [1‐^13^C]lactate (185 ppm) were used to calculate metabolite ratios. A quality control step was also applied for data display, where the acceptance threshold on the pyruvate signal amplitude was equivalent to a signal‐to‐noise ratio greater than 11 (intensity of a 50 Hz line‐width resonance divided by root‐mean‐squared noise). Voxels were selected for quantitative comparison between inflamed and non‐inflamed paws by drawing a region of interest around individual feet in the reference FSE image. Voxels whose centre co‐localized to this region of interest were included. A one‐way *t*‐test was performed on all voxels in a CFA‐inflamed paw, comparing the mean lactate‐to‐pyruvate ratio to that of both paws from a mouse with non‐inflamed paws. No correction was made for multiple comparisons.

### 2.6 MRS of hyperpolarized ^13^C‐labelled carbon dioxide and bicarbonate

Spectra were acquired from one non‐inflamed hind paw of four animals, 1 day prior to CFA injection. Spectra were similarly acquired, from four CFA‐inflamed paws in four animals five to seven days after CFA injection. Animals were culled following completion of the MR experiments. Mice were placed in the MR instrument with one foot (CFA‐inflamed or non‐inflamed, *n* = 4 feet for each group) pointing down through a 9 mm diameter custom‐built solenoid coil that covered the foot from heel to toes. Foot volumes were determined using the open‐source Fiji software package.^45^ The tissue was outlined in 2 mm thick proton image slices and the resulting areas for each slice were summed. The volume was calculated for the whole foot from the toes to the heel, where the top of the talus joins the leg. There was no significant difference in the body weights of mice with CFA‐inflamed and non‐inflamed paws at the time of imaging (mean 19 g). Carbon‐13 labelled caesium bicarbonate was prepared and polarized as described previously^40^, ^46^: 0.7 mmol of CsH^13^CO_3_ was dissolved in 0.54 mmol of glycerol (Sigma‐Aldrich) and 63 μl of water with 15 mmol/l OX063 and 1 mmol/l Dotarem. Hyperpolarized samples were dissolved in 6 ml superheated buffer containing 80 mM phosphate at pH 7.5 and 100 mg/l EDTA, and then rapidly ion exchanged with 3 g of Chelex 100 resin in the sodium form (Bio‐Rad Laboratories, Watford, UK) before injection via a tail vein. Pulse and acquire, coil‐localized, spectra (6000 Hz bandwidth, nominal flip angle of 10°, 0.45 ms echo time, 1024 data points) were acquired every second from 12 s after injection. The first 28 spectra—from 12 to 39 s—were summed, phase corrected and a quadratic baseline correction applied. The ^13^CO_2_/H^13^CO_3_ ratio was calculated by integrating the signal intensities between 127 and 123 ppm and between 163 and 156 ppm respectively and converted to a pH value by assuming a p*K*_a_ of 6.17.^40^

## 3 RESULTS

### 3.1 Significant hind‐paw inflammation is observable from 24 h after injection of CFA

**Figure 1:**
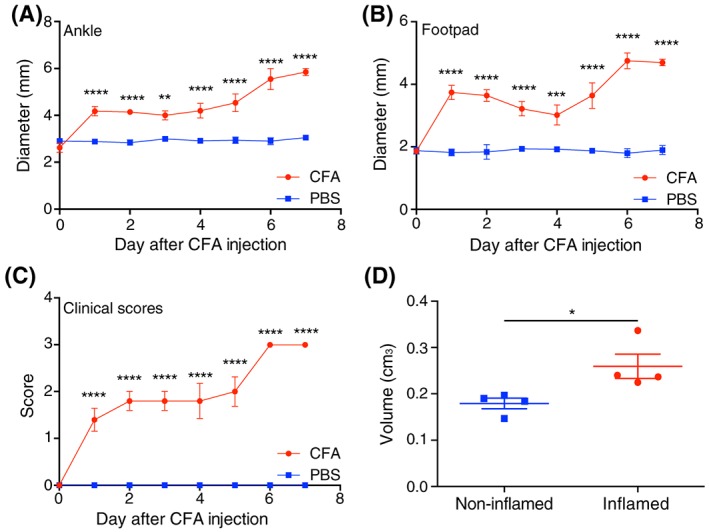
CFA‐induced inflammation. A, Ankle diameters of the hind paws of mice in the inflamed group that were used for pH measurements (*n* = 5 to Day 5; *n* = 2 to Day 7). The right paw was injected with CFA while the left was injected with PBS. B, Foot pad diameters of the same group of mice. C, Clinical scores for CFA‐ and PBS‐injected paws. D, Foot volumes estimated from ^1^H images of non‐inflamed and CFA‐inflamed paws at the time when pH measurements were made using hyperpolarized ^13^C‐labelled bicarbonate. *A one‐sided *t*‐test showed that the estimated volumes were significantly different between the two groups (*p* ≤ 0.05)

Inflammation of the hind paw induced by CFA injection can be followed by measuring paw swelling, as described by Chillingworth and Donaldson,^30^ and by observation of how swelling and joint damage produce anatomical changes in the foot, which is reflected in the clinical score.^42^ Following injection of CFA, inflammation of the ankle and footpad was observed within 24 h and was significantly greater than in PBS‐injected paws at all time points (Figure 1A,B, *p* < 0.0001, *n* = 5 mice). The clinical inflammation score also increased gradually from 24 h onwards, being significantly greater than in PBS‐injected paws at all time points (Figure 1C, *p* < 0.0001, *n* = 5 mice). These results are consistent with published data for this model^30^ and demonstrate that when the pH was measured there was significant inflammation in CFA‐injected mouse paws. These data were consistent with foot volumes estimated from MRI data. The CFA‐inflamed paws in which the pH was measured were significantly larger than non‐inflamed paws (non‐inflamed 0.180 ± 0.009 cm^3^ versus CFA inflamed 0.253 ± 0.021, *p* ≤ 0.01, Figure 1D, *n* = 4 feet). The mice used for the [1‐^13^C]pyruvate measurements showed similar significant swelling in CFA‐injected paws (data not shown).

### 3.2 Labelled lactate is elevated in the CFA‐inflamed paws of animals injected with hyperpolarized [1‐^13^C]pyruvate

**Figure 2:**
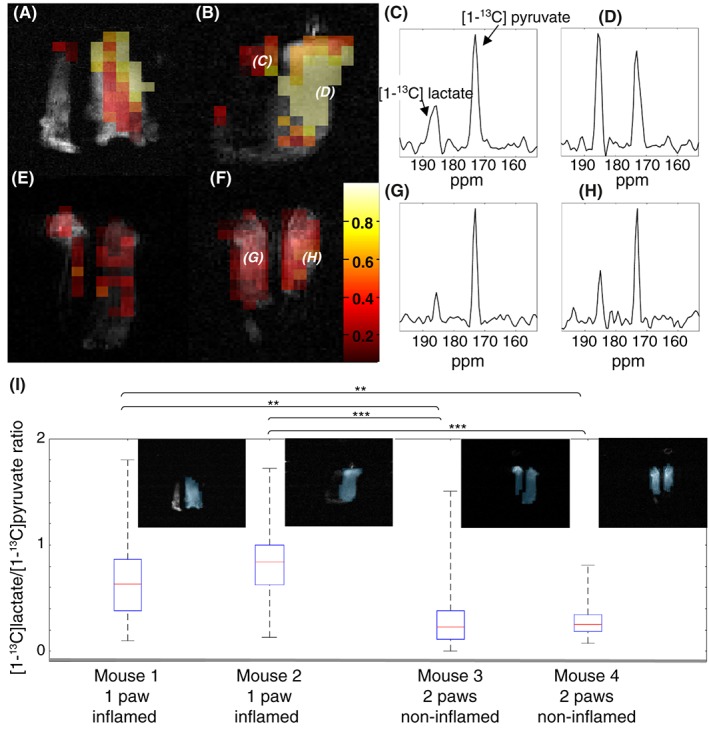
[1‐^13^C]lactate/[1‐^13^C]pyruvate ratios in animals injected with hyperpolarized [1‐^13^C]pyruvate. A,B, False colour images of the [1‐^13^C]lactate/[1‐^13^C]pyruvate ratio superimposed on the grey scale ^1^H image of tissue water in mice with one inflamed paw (right hand side of the image). Only those voxels where the pyruvate signal amplitude was more than 11 times the noise level are shown. C,D, Two voxels indicated in the ^13^C image shown in B are shown as MR spectra. E,F, False colour images of the [1‐^13^C]lactate/[1‐^13^C]pyruvate ratio superimposed on the grey scale ^1^H image of tissue water in mice with no inflammation. G,H, Two voxels indicated in the ^13^C image shown in F shown as MR spectra. The figure legend gives the colour scale for the lactate‐to‐pyruvate ratios between 0 (high pyruvate, no lactate) and 1 (equal lactate and pyruvate signal intensities). I, [1‐^13^C]lactate/[1‐^13^C]pyruvate ratios for all voxels within the inflamed hind paw in CFA‐injected mice (Mice 1 and 2) or both feet in mice with no inflammation (Mice 3 and 4). FSE ^1^H images (insets) show the positions of the ^13^C image voxels from which the [1‐^13^C]lactate/[1‐^13^C]pyruvate ratios were calculated. Differences between inflamed and normal paws were significant (**p < 10^−7^; ***p < 10^−16^, no corrections were made for multiple comparisons)

Seven days post‐CFA administration, ^13^C MRS images were acquired 20 s after i.v. injection of hyperpolarized [1‐^13^C]pyruvate. The conversion to [1‐^13^C]lactate was higher in CFA‐inflamed paws than in non‐inflamed paws. Although the images showed that there was a range of lactate‐to‐pyruvate ratios across the inflamed paw (Figure 2A‐D**)**, these were higher than in non‐inflamed paws (Figure 2E‐H) and in contralateral PBS‐injected paws (Figure 2C,D). Analysis of all voxels within CFA‐inflamed or non‐inflamed paws showed that there was a significant elevation of the lactate‐to‐pyruvate ratio in the CFA‐inflamed paws (Figure 2I). The mean lactate‐to‐pyruvate ratios were 0.30 ± 0.23 for non‐inflamed paws and 0.74 ± 0.32 for CFA‐inflamed paws (*p* < 0.001, for each individual comparison: see Figure 2I for details). This is consistent with increased levels of lactate^39^, ^47^, ^48^ and glycolysis in the inflamed tissue, where resident fibroblast‐like synoviocytes, which are key contributors to synovial inflammation, show a shift to glycolytic metabolism in RA.^49^

### 3.3 Measurements of the hyperpolarized [^13^C]carbon dioxide/[^13^C]bicarbonate ratio showed no significant difference in extracellular pH between CFA‐inflamed and non‐inflamed paws

**Figure 3:**
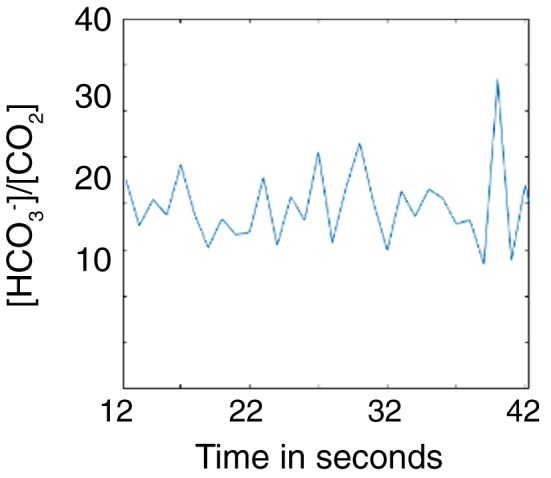
The ^13^C‐labelled bicarbonate/carbon dioxide signal ratios obtained from individual spectra acquired from one inflamed paw every second, from 12 s after ^13^C‐bicarbonate injection

**Figure 4:**
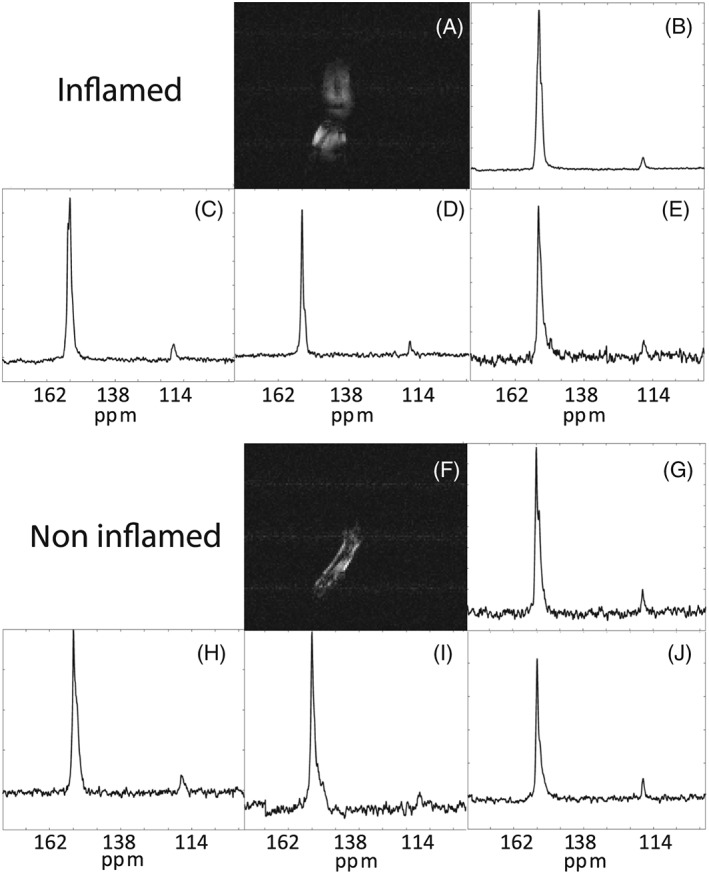
Measurement of ^13^C spectra after injection of ^13^C‐labelled bicarbonate in control and inflamed mouse paws. A, A gradient‐echo ^1^H MR image from a 2 mm thick slice through an inflamed mouse paw. B‐E, Summed ^13^C spectra acquired from four inflamed mouse paws between 12 and 39 s after injection of ^13^C‐labelled bicarbonate. The spectra show a larger ^13^C bicarbonate resonance and smaller carbon dioxide resonance. F, A gradient‐echo ^1^H MR image from a 2 mm thick slice through a normal mouse paw. G‐J, Summed ^13^C spectra acquired from four non‐inflamed mouse paws between 12 and 39 s after injection of ^13^C‐labelled bicarbonate

**Figure 5:**
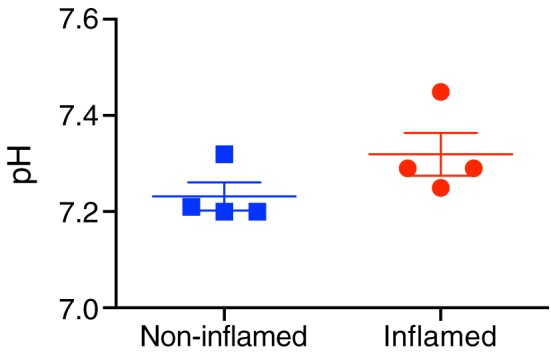
Individual pH values for four CFA‐inflamed and non‐inflamed right hind paws. A one sided *t*‐test of the null hypothesis that inflamed paws do not have lower pH than non‐inflamed paws was confirmed (*p* = 0.92)

To determine whether raised labelled lactate concentrations in the CFA model were accompanied by tissue acidosis we measured the ^13^CO_2_/H^13^CO_3_ ratio following i.v. injection of hyperpolarized [^13^C]bicarbonate. Serial spectra acquired from an individual animal showed that there was no change in the ^13^CO_2_/H^13^CO_3_^−^ ratio from 12 s after bicarbonate injection, demonstrating that the carbonic anhydrase‐catalysed conversion of bicarbonate to carbon dioxide had reached equilibrium and therefore that the pH could be estimated from this ratio (Figure 3). The ^13^C nuclear spin polarization in hyperpolarized H^13^CO_3_ and ^13^CO_2_ has a very short half‐life *in vivo* of only about 10 s,^40^ and by 39 s after bicarbonate injection the CO_2_ signal had decayed and was no longer detectable. Therefore, the interval between 12 and 39 s after bicarbonate injection was chosen for subsequent analysis, where spectra acquired during this period were summed in order to calculate a ^13^CO_2_/H^13^CO_3_^−^ ratio and a pH using the Henderson‐Hasselbach equation. Gradient echo ^1^H images show the increased size of a paw injected with CFA (compare Figure 4A with Figure 4F). Summed ^13^C spectra from CFA‐inflamed and non‐inflamed paws are shown in Figure 4B‐E and G‐J respectively, and the extracellular pH values calculated from the ^13^CO_2_/H^13^CO_3_ ratios in these spectra are shown in Figure 5. The extracellular pH values in CFA‐inflamed and non‐inflamed paws were not significantly different (pH 7.32 ± 0.09 versus pH 7.23 ± 0.06 respectively, *n* = 4, *p* = 0.92).

## 4 DISCUSSION

Although there have been several reports of tissue acidosis occurring in RA,^8^, ^9^, ^10^, ^11^ there is large individual variability.^9^, ^12^ In mouse models of arthritis it is often assumed that tissue acidosis occurs, but no data exists to substantiate this belief. We have shown here that intraplantar injection of CFA in the mouse paw induces inflammation and that this is accompanied by a significant increase in ^13^C lactate labelling in animals injected with hyperpolarized [1‐^13^C]pyruvate. However, despite the apparent increase in glycolytic activity and lactate production we were unable to find any evidence of extracellular acidification of this tissue, as assessed from measurements of the ^13^CO_2_/H^13^CO_3_ ratio in animals injected with hyperpolarized ^13^C‐labelled bicarbonate.

Measurements of lactate labelling in animals injected with hyperpolarized [1‐^13^C]pyruvate have been used previously to assess glycolytic activity in inflamed joints.^39^ MacKenzie et al.^39^ measured a [1‐^13^C]lactate/[1‐^13^C]pyruvate ratio of 0.52 ± 0.16 in CFA‐inflamed rat paws at 20 s after injection of hyperpolarized [1‐^13^C]pyruvate as compared with a value of 0.74 ± 0.32 (mean ± standard deviation from all image voxels of two inflamed feet, one each from two mice) measured here in CFA‐inflamed mouse paws, also at 20 s after injection. The considerable variation in the [1‐^13^C]lactate/[1‐^13^C]pyruvate ratio observed here (Figure 2), when compared with the rat model, reflects the small size of the mouse paw, particularly the non‐inflamed paw, which led to relatively low signal‐to‐noise ratios in the ^13^C spectra. Despite this, the images of control animals, without inflamed feet, showed consistent signal‐to‐noise ratios and [1‐^13^C]lactate/[1‐^13^C]pyruvate ratios throughout the feet. The variation in [1‐^13^C]lactate/[1‐^13^C]pyruvate ratio between CFA‐inflamed feet most likely reflects variation in local synovial hyperplasia and inflammatory cell invasion. While this measurement does not measure directly lactate concentration, it shows that the exchange of ^13^C label between pyruvate and lactate is faster. The exchange rate is, in part, dependent on the size of the regional lactate pool^47^, ^48^ and therefore is indicative of increased glycolytic activity in inflamed tissue. MacKenzie et al.^39^ showed marked infiltration of leukocytes in the majority of rats with CFA‐induced hind paw inflammation, with accumulation of inflammatory cells at sites of injection. Leukocytes become increasingly glycolytic on activation as do stromal cells, such as fibroblast‐like synoviocytes in RA.^49^ Increased glycolysis is consistent with previous studies that have shown this more directly, for example, synovial fluid taken from patients with RA has shown elevated lactate levels.^9^, ^12^, ^13^

The signal‐to‐noise ratios obtained following injection of hyperpolarized ^13^C‐labelled bicarbonate were not sufficient for imaging, and therefore for these experiments we acquired spectra from the whole mouse paw. Signal localization was obtained by placing a custom‐made receiver coil around the paw, the improved coil‐filling factor improving the signal‐to‐noise ratio. The measured pH corresponded therefore to that of the dominant tissue observed in MR images of this volume (see Figure 4), which was mainly muscle, tendon, ligament and connective tissue in the paw. The very short half‐life of the nuclear spin polarization in hyperpolarized H^13^CO_3_ and ^13^CO_2_ means that the ratio reflects predominantly the extracellular pH^46^, ^50^ in regions that are relatively well perfused and that would have also received the hyperpolarized [1‐^13^C]pyruvate. The absence of a decrease in extracellular pH measured here in CFA‐inflamed paws (pH 7.32 ± 0.09 versus pH 7.23 ± 0.06 in controls) is in contrast to a study by Scholz et al.,^41^ who used hyperpolarized [^13^C]bicarbonate to measure pH in an acute inflammation model in the rat leg (injection of concanavalin A 2 hours prior to imaging). The lower pH reported in this study (pH 7.0) may reflect the acute nature of the inflammation induced by concanavalin A, as compared with the more chronic inflammation induced by CFA, and also the later imaging time point used here. Our failure to observe a lowered pH could have been due to a lack of equilibration of hyperpolarized ^13^C label between H^13^CO_3_ and ^13^CO_2_, which is catalysed predominantly by carbonic anhydrase.^46^ However, we demonstrated that 12 s was sufficient to achieve label equilibration (Figure 3), which is less than the approximately 16 s required in a murine lymphoma *in vivo*,^49^ but comparable to the time required for label equilibration in rat heart muscle,^51^ which included the time for formation of carbon dioxide and bicarbonate from [1‐^13^C]pyruvate catabolism.

The hyperpolarized ^13^C‐labelled lactate detected following injection of hyperpolarized [1‐^13^C]pyruvate is predominantly intracellular, at least in animal tumour models,^52^ whereas the pH determined using hyperpolarized ^13^C‐labelled bicarbonate is predominantly extracellular.^40^ Therefore, our failure to detect a decrease in pH, despite an increase in glycolytic activity, may be because any pH decrease is largely intracellular. However, lactic acid is rapidly exported from cells,^53^, ^54^, ^55^ and it seems unlikely that there would not have been an increase in extracellular lactic acid concentration in the inflamed joints, in which case the resulting increase in H^+^ concentration must not have exceeded the extracellular buffering capacity of the tissue. Whatever the explanation, our results have shown that increased glycolytic activity in the inflamed joint is not accompanied by extracellular tissue acidosis.

Although we have not assessed pain behaviour here, this was inferred from measurements of joint swelling and worsening clinical scores, which are features that have been shown previously to correlate with indicators of pain, namely mechanical and thermal hyperalgesia, in CFA‐induced inflammation.^29^, ^30^, ^34^, ^56^ The results presented here would suggest, therefore, that tissue acidosis is not itself a primary contributor to the pain observed in the CFA‐induced arthritis model, which might perhaps explain the lack of relief from either mechanical or thermal hyperalgesia in mice lacking ASIC1, ASIC2 or ASIC3.^34^ Although TRPV1 knockout mice display diminished thermal hyperalgesia in the CFA model,^33^ this is likely due to a shift in the thermal sensitivity of TRPV1 activation resulting from inflammatory mediators such as nerve growth factor dependent removal of phosphatidylinositol 4,5‐bisphosphate inhibition of TRPV1,^57^ rather than due to acid‐mediated modulation of the TRPV1 thermal activation threshold, which can also occur.^58^

## 5 CONCLUSIONS

In summary, we have demonstrated that in the CFA‐inflamed mouse paw model of arthritis there is elevated production of lactate, but that this is not coupled with a significant decrease in extracellular pH. This result could explain the lack of phenotype observed in mice lacking different ASIC subunits and questions the validity of CFA‐induced arthritis as a model for RA, in which tissue acidosis has been demonstrated.

## CONFLICT OF INTEREST

The authors declare that they have no competing interests.

## AUTHOR CONTRIBUTIONS

AJW built the carbon solenoid coil, made hyperpolarized ^13^C reagents, designed and performed all DNP and MRI experiments, and wrote the initial draft. ZMAH and GC carried out CFA experiments and made daily measurements of inflammation; DH assisted with MRI experiments; KMB and EStJS managed the project and edited the manuscript. All authors approved the final manuscript.
